# On the utilization of deep and ensemble learning to detect milk adulteration

**DOI:** 10.1186/s13040-019-0200-5

**Published:** 2019-07-08

**Authors:** Habib Asseiss Neto, Wanessa L.F. Tavares, Daniela C.S.Z. Ribeiro, Ronnie C.O. Alves, Leorges M. Fonseca, Sérgio V.A. Campos

**Affiliations:** 1 Federal Institute of Mato Grosso do Sul, Rua Ângelo Melão, 790, Três Lagoas, 79641-162 MS Brazil; 2 Department of Computer Science, Federal University of Minas Gerais, Av. Antônio Carlos, 6627, Belo Horizonte, 31270-901 MG Brazil; 3 Veterinary School, Federal University of Minas Gerais, Av. Antônio Carlos, 6627, Belo Horizonte, 31270-901 MG Brazil; 4 Instituto Tecnológico Vale, R. Boaventura da Silva, 955, Belém, 66055-090 PA Brazil; 5 Federal University of Pará, R. Augusto Corrêa, 1, Belém, 66075-110 PA Brazil

**Keywords:** Classification, Machine learning, Deep learning, Ensemble learning, Infrared spectroscopy, Milk, Adulteration

## Abstract

**Background:**

Fraudulent milk adulteration is a dangerous practice in the dairy industry that is harmful to consumers since milk is one of the most consumed food products. Milk quality can be assessed by Fourier Transformed Infrared Spectroscopy (FTIR), a simple and fast method for obtaining its compositional information. The spectral data produced by this technique can be explored using machine learning methods, such as neural networks and decision trees, in order to create models that represent the characteristics of pure and adulterated milk samples.

**Results:**

Thousands of milk samples were collected, some of them were manually adulterated with five different substances and subjected to infrared spectroscopy. This technique produced spectral data from the milk samples composition, which were used for training different machine learning algorithms, such as deep and ensemble decision tree learners. The proposed method is used to predict the presence of adulterants in a binary classification problem and also the specific assessment of which of five adulterants was found through multiclass classification. In deep learning, we propose a Convolutional Neural Network architecture that needs no preprocessing on spectral data. Classifiers evaluated show promising results, with classification accuracies up to 98.76%, outperforming commonly used classical learning methods.

**Conclusions:**

The proposed methodology uses machine learning techniques on milk spectral data. It is able to predict common adulterations that occur in the dairy industry. Both deep and ensemble tree learners were evaluated considering binary and multiclass classifications and the results were compared. The proposed neural network architecture is able to outperform the composition recognition made by the FTIR equipment and by commonly used methods in the dairy industry.

**Electronic supplementary material:**

The online version of this article (10.1186/s13040-019-0200-5) contains supplementary material, which is available to authorized users.

## Background

Milk fraudulent adulteration consists of adding foreign substances to the milk. This is a common practice in Brazil and several countries worldwide [12], with the objective of increasing the product volume, disguising poor quality parameters and profiting with illegal actions [2, 7, 22]. Different substances can be added to milk with specific purposes. For instance, sucrose and starch are often used to modify density and freezing point after extra water added to milk. Sodium bicarbonate can be added to reduce high acidity levels related to high bacteria contamination and bad manufacturing practices. Hydrogen peroxide and formaldehyde can preserve microbial count related to poor milk quality [9].

Fourier Transformed Infrared spectroscopy (FTIR) is one of the most commonly used techniques to read the composition of a sample in the food industry [9]. FTIR is a fast, nondestructive, and simple method that can be applied for milk composition analysis and it generates spectral data that can be computationally explored [22]. Machine learning techniques provide ways of understanding spectral data and producing useful knowledge regarding milk composition quality for consumers and regulatory agencies.

These techniques have been widely used in several areas and classification is a common machine learning task capable of understanding and categorizing data. In supervised learning, the classification task involves a training process with labeled data in order to generate a computational model that learns with that data [14]. Once the model is trained, it can be used to predict the label of new, unseen data. In the testing process, the model can be applied to a dataset and predicted labels can be compared to actual labels. Then, classification accuracy is used to evaluate the predictive capabilities of the model [10]. Deep and ensemble learners are two well-known methods with different characteristics that have shown excellent performance in several machine learning applications.

*Ensemble learners* are methods that combine many models’ predictions. Bagging (Bootstrap Aggregating) is a technique that trains several machine learning models independently with randomly chosen subsets of data, and it uses majority voting for aggregating the outputs of base learners [14]. Boosting also trains classifiers using different training sets, but they are learned sequentially, with each model trying to minimize the error from the previous one. The combination of individually weak learners creates a better performing model [10]. Random Forest (RF) and Gradient Boosting Machine (GBM) are examples of bagging and boosting techniques, respectively. Since ensemble methods rely on the combination of models, they build smooth decision boundaries capable of finding the optimal feature and model combination to the classification problem [21].

In the area of *deep learning*, Convolutional Neural Networks (CNNs) are gaining great attention due to their high accuracy in pattern recognition and it has been successfully applied in a diversity of classification problems [19]. When compared to regular neural networks, additional layers (convolutional layers) are used in CNNs in order to filter input data and learn specific features from the data with different levels of abstraction [17].

Machine learning classifiers have been applied successfully in many applications, including image recognition, speech detection, and signal processing. Considering spectral data classification, decision trees have been used for classification of landscapes using satellite spectral data [8]. CNNs have been applied to electrocardiogram signals (ECG), significantly outperforming other ECG classification methods [16]. Mineral spectrum classification using CNN has achieved interesting results and has been compared to other machine learning methods [17]. CNNs also have been applied to audio spectral data for detecting sound events with human-level accuracy [15].

Milk adulteration analysis has been done with more traditional statistical methods, such as Principal Component Analysis (PCA) and Partial Least Squares (PLS) regression, that have been applied to infrared spectroscopy data in order to obtain adulteration estimates of whey, synthetic milk, hydrogen peroxide and others [22]. Milk adulteration by whey has also been studied by measuring specific proteins using PCA from spectral data [7]. Different milk adulterants have been analyzed with infrared spectroscopy using PCA multivariate analysis [9]. The work from [2] has similarities with our study by also using neural networks for milk adulteration detection. However, the authors used a regression model for quantifying the adulteration by a single ingredient (whey).

The objective of this work was to perform experiments with classification methods to recognize patterns in infrared milk composition in order to predict possible adulterations by foreign substances.

## Methods

In this work, the characterization of bovine milk was made using machine learning techniques to detect the presence of milk adulterants or to assert which adulterant was found. In order to accomplish this, classification methods were used to determine milk sample adulteration. Classical statistical learning strategies such as Logistic Regression, Linear Regression, and PLS, usually employed by the industry [6, 7], were explored as benchmark models. Ensemble and deep learning classifiers were trained and tested on real, manually adulterated, milk samples in order to recognize patterns that identify adulteration characteristics.

Two versions of the classification problem were considered: binary and multiclass classifications. In the binary problem, the possible classes for a sample classification were either the presence or absence of an adulterant. In the multiclass problem, the classes were either one of the specific adulterant added to the milk or the “raw” class, when the sample has no adulterant added.

### Data acquisition and sample preparation

Milk samples were acquired from the experimental farm at the Federal University of Minas Gerais, Brazil, and from the Laboratory for Milk Quality Analysis (Accredited ISO/IEC 17025) at the same university using commercial milk samples from the laboratory routine processes. A total of 4846 milk samples were collected, whereas 2376 were adulterated for the purpose of this study. The adulterated milk samples were added with one of five different substances (all of analytical grade): sucrose, soluble starch (amylose and amylopectin), sodium bicarbonate, hydrogen peroxide, and formaldehyde (Synth, Brazil). Although multiple adulterants can be found at once in a fraudulent milk sample [4, 24], in this work we aimed to analyze the effects of each adulterant individually, in order to describe how it affects pure milk composition.

FTIR spectroscopy was applied to all the collected milk samples in order to obtain infrared spectra, using the FTIR equipment (LactoScope™ FTIR 400, Delta Instruments, Drachten, The Netherlands), which outputs two pieces of information for each analyzed sample: an infrared spectrum file (SPC format) that contains coordinates for the infrared spectrum and a components file (CSV format), which contains numerical variables, called component features, that the equipment calculates from the infrared spectrum Additional file 2.

**Fig. 1:**
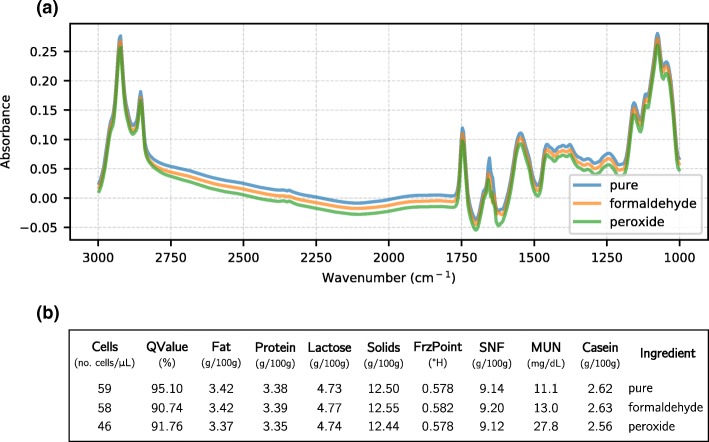
**a** Plot of the infrared spectra for three randomly selected samples of the classes pure, formaldehyde and peroxide, acquired by the FTIR equipment. The raw spectra were analyzed directly by the proposed Convolutional Neural Network. Each spectrum was plotted with subtle shifts for viewing purpose. **b** Component features for the same samples, generated by the FTIR equipment and stored in CSV format. Each column quantifies an important milk composition information. The columns for fat, protein, lactose, solids, solids non-fat (SNF), casein and milk urea nitrogen (MUN) represent each component concentration in the sample. The Cells column represents the somatic cells counting, FrzPoint represents the freezing point of the sample, with values given in degrees Hortvet (^∘^H), and QValue is a calculation of the sample quality by the equipment. These numerical features were analyzed by ensemble methods

In our milk dataset, each sample is represented by both the component features and the spectral data. However, we used each of the two types of data differently. The component features data structure is ideal for the application of a decision tree classifier because each feature strongly represents some known characteristics in the milk composition. Since the combination of several classifiers may reduce the risk of an unfortunate selection of a poorly performing classifier [21], ensemble tree learners were chosen for this task. On the other hand, the spectral data are composed by the full spectral coordinates, which can be interpreted as “images” for neural network recognition. For the latter, we used CNNs that are capable of detecting specific features from spectra without any required preprocessing. In Fig. 1 we show some spectra and some extracted component features.

For the purpose of estimating the quality of our classifiers, the hold-out cross-validation technique [3] was performed with three pairs of training/testing subsets with proportions: 90/10%, 75/25%, and 50/50%. This was the preferred split strategy since the same subsets needed to be tested with different classifiers, including deep learning, that usually splits datasets into training/validation/test sets. Each subset was obtained randomly from the original dataset (4846 samples) and the class distribution remained: ≈50% for raw milk and ≈10% for each of the five adulterant classes. Dataset samples distribution is described in Table 1. Detailed class distributions for each training and test dataset split are presented in Table 2.

**Table 1:** Sample distribution for the binary and multiclass versions of the collected dataset

Multiclass	# Samples	Binary	# Samples
Raw	2470	Raw	2470
Sucrose	486	Adulterated	2376
Formaldehyde	485		
Starch	480		
Peroxide	465		
Bicarbonate	460		

The full dataset has 4846 samples. The raw class is ≈50% of the dataset. Each adulterant class is ≈10% on multiclass, while the binary considers these samples as one class (≈50%)

**Table 2:** Class distribution for samples in each split of training and test set in multiclass version

Dataset split	Classes	# Training samples	# Test samples
90/10%	Raw	2213	257
	Bicarbonate	419	41
	Formaldehyde	442	43
	Peroxide	417	48
	Starch	439	41
	Sucrose	431	55
75/25%	Raw	1846	624
	Bicarbonate	338	122
	Formaldehyde	347	138
	Peroxide	364	101
	Starch	359	121
	Sucrose	380	106
50/50%	Raw	1239	1231
	Bicarbonate	219	241
	Formaldehyde	223	262
	Peroxide	242	223
	Starch	253	227
	Sucrose	247	239

In binary version, the five classes of adulterant substances are summed up as one “adulterated” class

### Analysis of component features using ensemble learners

During the process of reading the infrared spectrum, the FTIR equipment performs a series of calculations that determines numerical values for different milk components. According to the equipment documentation, calculations are based on a Multiple Linear Regression (MLR) model that considers the absorbance of light energy by the sample for specific wavelength regions. The extracted information depends on the equipment calibrations for milk components concentration (fat, protein, lactose, total solids, solids non-fat/SNF, casein and milk urea nitrogen/MUN). Other three extra values are also included: the somatic cells counting, the freezing point value and a quality control value (Q-Value).

**Fig. 2:**
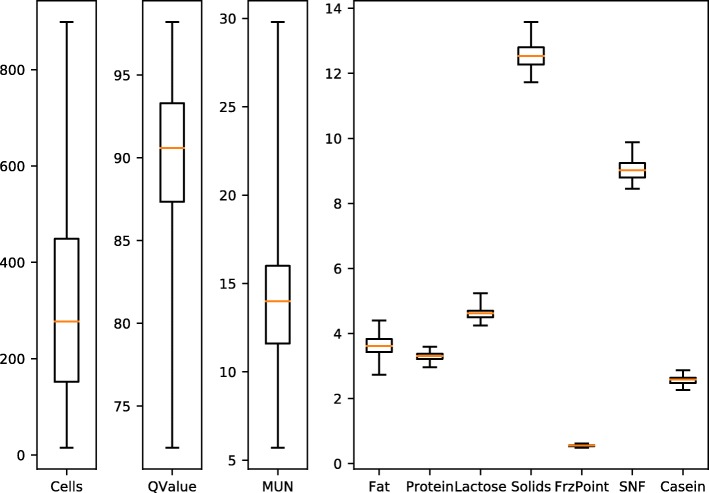
Boxplot of the component features from the dataset, analyzed by ensemble learners. The plots show scales and variation of each feature. Cells, QValue and MUN have significantly different scales and were plotted separately from the other variables. The Cells plot show that most samples has somatic cells counting from ≈150 to ≈450. The QValue plot shows that most samples on the dataset were considered of high quality (≈87 to ≈93). The FrzPoint plot shows that all samples has a freezing point at just above zero. All the other plots consider specific component concentrations and they show low variation

Pairwise correlations were calculated on standardized variables from the dataset. The relationship among these variables demonstrated that protein and casein are highly correlated (0.96). Since casein is a specific milk protein, the correlation makes sense. Correlations were also found with solids and fat (0.85), lactose with freezing point (0.77), and lactose with SNF (0.81). Other variables were found to be not expressively correlated. The complete feature correlation is presented in Additional file 1: Figure S1. All variables were read from equipment generated CSV file and were used as features in ensemble decision tree learners. The adulterants added to each sample were considered class labels for the samples and were used for training the classifiers. Figure 2 shows a boxplot considering scale and variation of all component features.

The component features were analyzed with Random Forest and Gradient Boosting Machine ensemble learners using the default implementations available in Scikit-learn [20]. The number of learners is controlled by the parameter n_estimators and it was set as 200 for each classifier. Models from both methods were evaluated for each available training and test sets using component features present in the samples. Binary and multiclass classifications were performed considering the same datasets.

### Analysis of infrared spectra using deep learning

The infrared spectra used as input to the CNN classifier were produced by the FTIR technique. They are formed by 518 points measured in wavenumbers ranging from 3000 cm^-1^ to 1000 cm^-1^. In the dataset, each spectrum is followed by the class label (adulteration substance), which allows the network to be trained. During the training process, the convolutional layers are used as filters that recognize specific features within spectral regions. For that reason, CNNs are able to receive raw spectral data as input, without the need of any preprocessing step, and they can handle important feature extraction from the data with no manual interaction [23].

**Fig. 3:**
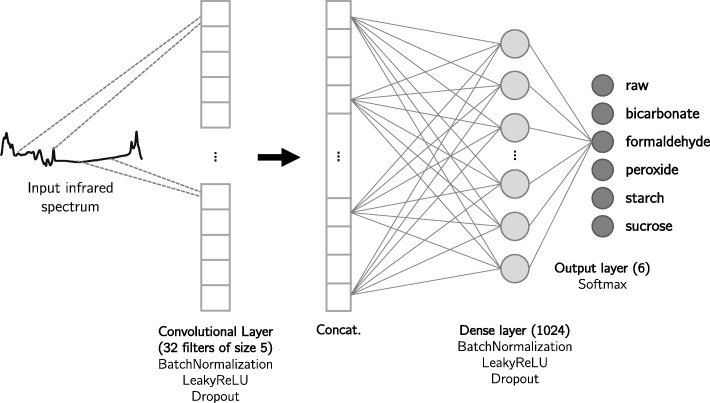
The proposed Convolutional Neural Network for multiclass classification of whole infrared spectra. The architecture consists of one convolutional layer that learns 32 filters of kernel size 5, which is capable of recognizing features directly from the raw infrared spectra. The output of the convolutional layer is concatenated then passed as input to a dense (fully-connected) layer, consisting of 1024 neurons. BatchNormalization, LeakyReLU and Dropout operations are performed in both convolutional and dense layers. Finally, the output layer of size 6 (the number of classes in multiclass problem) is activated by the Softmax function

We propose a CNN architecture that has one 1-dimensional convolutional layer that learns 32 filters of kernel size 5, which are capable of extracting features directly from the infrared spectra. Filters are concatenated and followed by one dense (fully connected) layer of 1024 neurons. At each layer, LeakyReLU [18] activation is used to add non-linearity to the model. Batch normalization [11] and dropout operations [25] are also performed at each layer so that the model avoids overfitting to the training data. The proposed network structure was based on the work from [17] but our structure is much simpler, with fewer layers and filters. In Fig. 3 we show the proposed CNN architecture.

For binary and multiclass classifications, we trained a CNN that differed only at the number of neurons in the output layer. Since this layer outputs the classification, the number of neurons must be exactly the number of classes we want to classify our data. So, the CNN for the binary classification has an output layer of one neuron with binary output, activated by the sigmoid function and the CNN for the multiclass classification has an output layer of six neurons, activated by the softmax function [26]. The binary model classifies the samples with the presence or absence of an adulterant and the multiclass classification classifies samples as raw milk or one of five known adulterant substances.

**Fig. 4:**
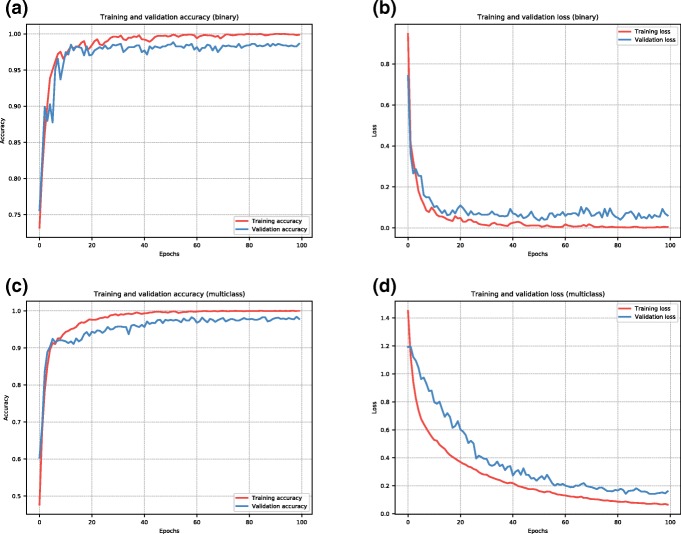
Plot of the CNN model’s accuracy and loss on training and validation steps considering the dataset split 80%/20%. The model was trained for 100 epochs. **a** Accuracy of training and validation considering the binary problem. **b** Loss of training and validation considering the binary problem. **c** Accuracy of training and validation considering the multiclass problem. **d** Loss of training and validation considering the multiclass problem. Each plotted curve is obtained from the history of the Keras model, which calculates both accuracy and loss for each epoch performed by the network. Accuracy is calculated by comparing the predicted class to the actual class. Loss is calculated by the cross entropy value between the predicted class and the actual class

The CNN training was made using Adam optimizer [13] for 100 epochs for both binary and multiclass problems. Every CNN execution considered 20% of the training set as the validation set. Figure 4 shows plots for the model’s accuracy and loss of training and validation sets. The plots show that validation of the network achieved better results in the binary problem when compared to the multiclass problem, which is expected because the binary is considered a simpler problem. The CNN architecture was implemented in Keras [5] and TensorFlow [1] in Python. All CNN processing was made on a personal laptop computer. The model training takes up to 16 min, while the classification for all samples in the test dataset takes at most 270 ms.

## Results and discussion

In order to determine that the chosen techniques and machine learning models were adequate for our experiments, we conducted a test that compared the performance of methods that are simpler and more commonly used in the dairy industry: Logistic Regression, Linear Regression and PLS [6, 7]. Classification versions of these methods were evaluated for each dataset split with whole spectra using the default implementations available in Scikit-learn. Accuracies for Logistic Regression ranged from 55.92% to 58.76% in multiclass and from 71.40% to 76.49% in binary classification. For Linear Regression, accuracies ranged from 31.55% to 33.50% in multiclass and 79.20% to 79.62% in binary classification. Finally, for PLS, accuracies ranged from 32.56% to 35.26% in multiclass and 76.91% to 77.39% in the binary problem. Although all methods had relatively good performances in the binary problem, accuracies were not satisfactory in multiclass classifications. Therefore, these values serve as a comparative basis for our ensemble and deep learners. Table 3 shows all accuracy values from Linear Regression, Logistic Regression, and PLS models.

**Table 3:** Accuracy from simpler classifiers (Logistic Regression, Linear Regression, and Partial Least Squares) for binary and multiclass classifications that serve as baseline for our deep and ensemble learners

Dataset	Classification	Logistic Regression	Linear Regression	Partial Least Squares
90/10%	Multiclass	0.5876	0.3155	0.3526
	Binary	0.7649	0.7959	0.7691
75/25%	Multiclass	0.5693	0.3350	0.3267
	Binary	0.7583	0.7962	0.7739
50/50%	Multiclass	0.5592	0.3281	0.3256
	Binary	0.7140	0.7920	0.7714

All classifiers were evaluated with 3 pairs of training and test datasets randomly selected from our milk samples, identified by their proportion of training and test samples

Both ensemble and deep learners were evaluated for adulterant detection on milk samples. The dataset has 4846 samples labeled as one of six possible classes: raw, sucrose, starch, bicarbonate, peroxide, and formaldehyde. For the multiclass version of the problem, all six classes were used. For the binary version, classes for each adulterant were considered as one class: adulterant present, while class raw was considered as the second class. Binary and multiclass classifications were evaluated with the selected subsets of training and testing described earlier for GBM, RF, and CNN classifiers. For the ensemble methods, classification accuracies ranged from 86.09% to 98.56%. The proposed CNN produced accuracies up to 98.76%. The mean accuracies for RF, GBM and CNN were 93.23%, 92.25% and 96.76%, respectively. Accuracy values show that all classifiers have better performance on binary classifications. However, CNN has shown to be a more robust classifier, since it has very close accuracy levels with both binary and multiclass problems. All accuracy results from our models are shown in Table 4. We also detail the accuracy results per class in multiclass classifications for RF, GBM and CNN in Table 5. These values show that all classifiers have better performance on ‘raw’ classification, and that CNN has a best overall performance with every class. Values also show that increasing the training set (i.e., 90%) not always leads to better predictive performance in all classes. Finally, the CNN classifier is generally more robust when there is a decrease in training test size (50%).

**Table 4:** Accuracy from evaluated classifiers (RF, GBM, and CNN) for binary and multiclass classifications

Dataset	Classification	RF	GBM	CNN
90/10%	Multiclass	0.9093	0.8907	0.9608
	Binary	0.9856	0.9711	0.9794
75/25%	Multiclass	0.8812	0.8787	0.9695
	Binary	0.9744	0.9686	0.9876
50/50%	Multiclass	0.8700	0.8609	0.9538
	Binary	0.9736	0.9653	0.9546

All classifiers were evaluated with 3 pairs of training and test datasets randomly selected from our milk samples, identified by their proportion of training and test samples

**Table 5:** Accuracies for each individual class (bicarbonate, formaldehyde, peroxide, raw, starch, and sucrose) for multiclass classifications considering RF, GBM and CNN classifiers, in each of the selected training and test datasets: 90/10%, 75/25%, and 50/50%

Classifier	Dataset	Bicarbonate	Formaldehyde	Peroxide	Raw	Starch	Sucrose
RF	90/10%	0.7804	0.7209	0.7916	0.9883	0.8048	0.9636
	75/25%	0.7540	0.6884	0.7524	0.9839	0.8099	0.8773
	50/50%	0.7634	0.6259	0.7847	0.9805	0.7444	0.8953
GBM	90/10%	0.7804	0.7441	0.7291	0.9766	0.7560	0.9272
	75/25%	0.8032	0.6739	0.7623	0.9759	0.7768	0.8867
	50/50%	0.7551	0.6259	0.7309	0.9780	0.7356	0.8870
CNN	90/10%	0.9756	0.9302	0.8958	0.9844	0.9024	0.9636
	75/25%	0.9918	0.9057	0.9108	0.9887	0.9421	1.0000
	50/50%	0.9958	0.9236	0.8340	0.9861	0.8854	0.9539

**Fig. 5:**
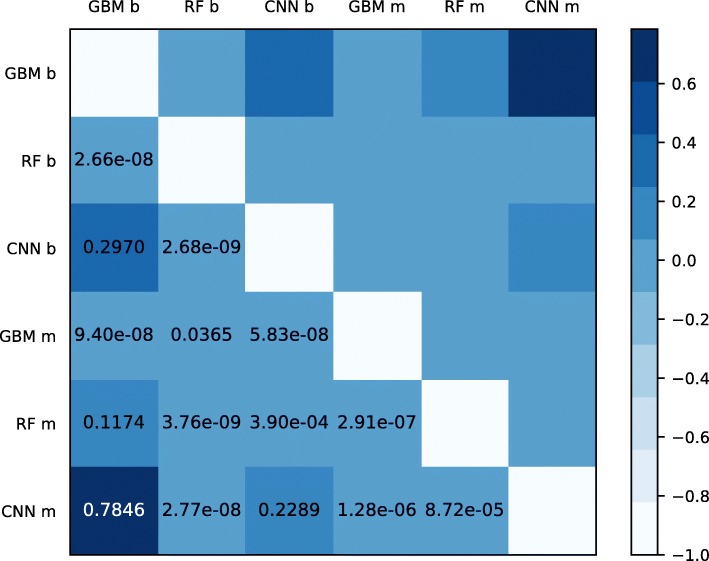
A t-test over pairwise differences in average AUC score for binary (**b**) and multiclass (**m**) versions of GBM, RF and CNN classifiers

**Fig. 6:**
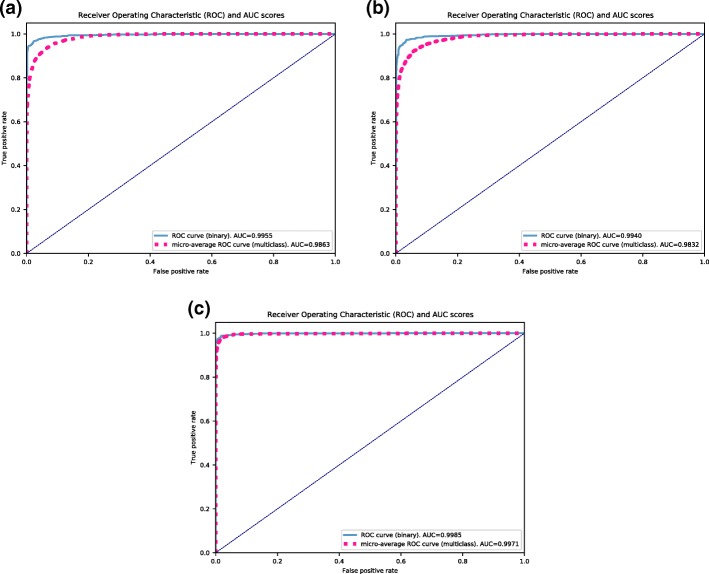
ROC (Receiver Operating Characteristic) curves for binary and multiclass versions of (**a**) Random Forest, (**b**) Gradient Boosting Machine and (**c**) Convolutional Neural Network and their AUC (Area Under the ROC Curve) score. Multiclass ROC was calculated using micro-average strategy, that sums up the individual true positives, false positives, and false negatives for all classes

The area under the ROC (Receiver Operating Characteristic) curve was evaluated for five repetitions in all classifiers, which yielded the AUC score. We then performed a pairwise t-test (t-value) comparing the difference in average AUC score across classifiers for binary and multiclass classifications. The greater the magnitude of *t*, the greater the evidence against the null hypothesis. This means there is greater evidence that there is a significant difference. The closer *t* is to 0, the more likely there isn’t a significant difference. The larger the absolute value of the t-value, the smaller the p-value, and the greater the evidence against the null hypothesis. Statistical significance tests show that the CNN classifiers are more robust, having significant differences in performance when compared to ensemble ones, as shown in Fig. 5. The ROC curves are presented in Fig. 6, where it is shown that all ROC curves from binary classification (continuous lines) show good performance and predictive power, while the multiclass ROC curves (dotted lines) show that the CNN model has better predictive performance.

Intuitively, the binary classification tends to be a simpler problem and can lead to better results, which is observed on the RF and GBM results, where binary classifications accuracies are at most 10% higher than multiclass accuracies. However, the CNN results show that the method is more robust on the multiclass classifications, with accuracies slightly lower than binary versions. We conclude that CNNs are particularly better suited for multiclass classification in this problem.

It is important to notice that the number of adulterated milk samples in our dataset is roughly half the total samples, which in terms of binary classification leads to balanced class distribution. On the other hand, when it comes to multiclass classification, we have six different classes and the majority of samples are of type raw, which leads to imbalanced class distribution. However, our method showed the capability to handle this situation without any issues, as shown in Table 4.

## Conclusion

In this work, we investigated milk composition and performed adulterant detection on FTIR spectral data by classifying samples using deep and ensemble tree learners. We collected 4846 milk samples and manually adulterated 2376 samples, using different classifiers to train models that are capable of recognizing composition characteristics that adulterants cause in milk. The classification was performed using two types of data: the whole infrared spectra analyzed by CNN and the 10 component features extracted from the spectra analyzed by RF and GBM classifiers.

Both methods, whole infrared spectra analyzed by CNN and the ten component features extracted from the spectra analyzed by RF and GBM classifiers achieved high accuracy, however, the CNN obtained better results, which is intuitive since it uses a more dense dataset (spectral coordinates). In other words, the extraction of the components performed by the FTIR equipment is not as representative as the features recognized by the proposed CNN architecture. Classification accuracies range from 86.09% to 98.76%.

Nevertheless, some challenges remain as future work, like a more profound study on the models’ interpretability, such as feature importance analysis and variable interactions. New analyses with multiple adulterations per sample and their effects on milk composition are also considered. Finally, we consider as an extension of this work a metaclassifier application, where the predictions of the deep and ensemble models could be combined, potentially achieving better performances.

## Additional files

Additional file 1 **Figure S1**. On the utilization of deep and ensemble learning to detect milk adulteration. (PDF 56 kb)

Additional file 2 A dataset containing nearly 1000 readings from milk samples in the CSV format. FTIR component features and spectral data points are provided for each sample. (CSV 9075 kb)

## Data Availability

A sample of the dataset used for this study is included in the Additional file 1 accompanying this article.

## Abbreviations

AUC: Area under the ROC curve
CNN: Convolutional neural network
ECG: Electrocardiogram
FTIR: Fourier transformed infrared
GBM: Gradient boosting machine
MLR: Multiple linear regression
MUN: Milk urea nitrogen
PCA: Principal component analysis
PLS: Partial least squares
RF: Random forest
ROC: Receiver operating characteristic
SNF: Solids non-fat
