# Supplementary material for: On the utilization of deep and ensemble learning to detect milk adulteration
Source: BioData Min. 2019 Jul 8;12:13. doi: 10.1186/s13040-019-0200-5 (PMC6615233; doi:10.1186/s13040-019-0200-5)
Supplement: Supplementary file 1 — Figure S1. On the utilization of deep and ensemble learning to detect milk adulteration. (PDF 56 kb) [file 13040_2019_200_MOESM1_ESM.pdf]

On the utilization of deep and ensemble learning to detect milk adulteration

Supplementary Material

Supplementary Figure

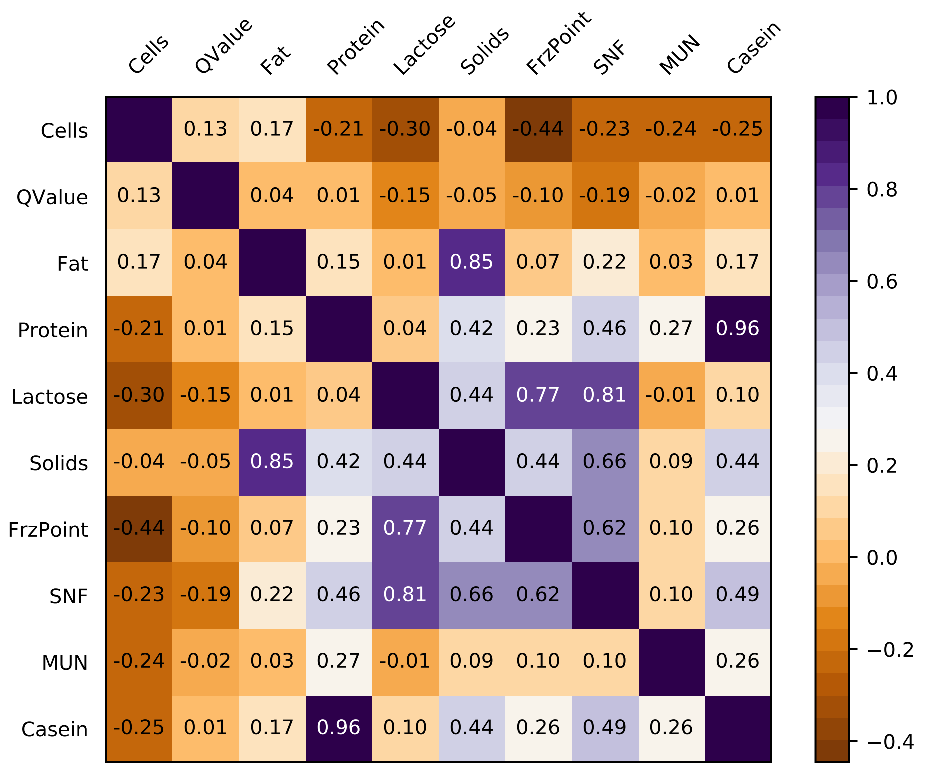

Fig. S1: Milk component features correlation matrix calculated using the Pearson correlation coefficient. Calculations were performed on standardized variables from the dataset. The values indicate that casein and protein are highly correlated, since casein is a type of protein in milk. Solids and fat are also correlated, since fat is a solid milk component. Lactose is correlated with freezing point and solids-not-fat (SNF). Other variables are not significantly correlated.
